# Percutaneous vs. surgical revascularization of non-ST-segment elevation myocardial infarction with multivessel disease: the SWEDEHEART registry

**DOI:** 10.1093/eurheartj/ehae700

**Published:** 2024-11-27

**Authors:** Elmir Omerovic, Truls Råmunddal, Petur Petursson, Oskar Angerås, Araz Rawshani, Sandeep Jha, Kristofer Skoglund, Moman A Mohammad, Jonas Persson, Joakim Alfredsson, Robin Hofmann, Tomas Jernberg, Ole Fröbert, Anders Jeppsson, Emma C Hansson, Göran Dellgren, David Erlinge, Björn Redfors

**Affiliations:** Department of Cardiology, Sahlgrenska University Hospital, Gothenburg, Sweden; Department of Molecular and Clinical Medicine, Institute of Medicine, Sahlgrenska Academy, University of Gothenburg, Gothenburg, Sweden; Department of Cardiology, Sahlgrenska University Hospital, Gothenburg, Sweden; Department of Molecular and Clinical Medicine, Institute of Medicine, Sahlgrenska Academy, University of Gothenburg, Gothenburg, Sweden; Department of Cardiology, Sahlgrenska University Hospital, Gothenburg, Sweden; Department of Molecular and Clinical Medicine, Institute of Medicine, Sahlgrenska Academy, University of Gothenburg, Gothenburg, Sweden; Department of Cardiology, Sahlgrenska University Hospital, Gothenburg, Sweden; Department of Molecular and Clinical Medicine, Institute of Medicine, Sahlgrenska Academy, University of Gothenburg, Gothenburg, Sweden; Department of Cardiology, Sahlgrenska University Hospital, Gothenburg, Sweden; Department of Molecular and Clinical Medicine, Institute of Medicine, Sahlgrenska Academy, University of Gothenburg, Gothenburg, Sweden; Department of Cardiology, Sahlgrenska University Hospital, Gothenburg, Sweden; Department of Molecular and Clinical Medicine, Institute of Medicine, Sahlgrenska Academy, University of Gothenburg, Gothenburg, Sweden; Department of Cardiology, Sahlgrenska University Hospital, Gothenburg, Sweden; Department of Molecular and Clinical Medicine, Institute of Medicine, Sahlgrenska Academy, University of Gothenburg, Gothenburg, Sweden; Department of Cardiology, Clinical Sciences, Lund University, Skåne University Hospital, Lund, Sweden; Karolinska Institutet, Department of Clinical Sciences, Danderyd University Hospital, Division of Cardiovascular Medicine, Stockholm, Sweden; Department of Health, Medicine and Caring Sciences, Linköping University, Linköping, Sweden; Department of Cardiology, Linköping University, Linköping, Sweden; Department of Clinical Science and Education, Division of Cardiology, Karolinska Institutet, Södersjukhuset, Stockholm, Sweden; Karolinska Institutet, Department of Clinical Sciences, Danderyd University Hospital, Division of Cardiovascular Medicine, Stockholm, Sweden; Department of Clinical Medicine, Faculty of Health, Aarhus University, Aarhus, Denmark; Department of Cardiology, Örebro University Hospital, Örebro, Sweden; Department of Molecular and Clinical Medicine, Institute of Medicine, Sahlgrenska Academy, University of Gothenburg, Gothenburg, Sweden; Department of Cardiothoracic Surgery, Sahlgrenska University Hospital, Gothenburg, Sweden; Department of Molecular and Clinical Medicine, Institute of Medicine, Sahlgrenska Academy, University of Gothenburg, Gothenburg, Sweden; Department of Cardiothoracic Surgery, Sahlgrenska University Hospital, Gothenburg, Sweden; Department of Molecular and Clinical Medicine, Institute of Medicine, Sahlgrenska Academy, University of Gothenburg, Gothenburg, Sweden; Department of Cardiothoracic Surgery, Sahlgrenska University Hospital, Gothenburg, Sweden; Department of Cardiology, Clinical Sciences, Lund University, Skåne University Hospital, Lund, Sweden; Department of Cardiology, Sahlgrenska University Hospital, Gothenburg, Sweden; Department of Molecular and Clinical Medicine, Institute of Medicine, Sahlgrenska Academy, University of Gothenburg, Gothenburg, Sweden

**Keywords:** Non-ST-segment elevation myocardial infarction, Revascularization, Multivessel disease, Percutaneous coronary intervention, Coronary artery bypass grafting

## Abstract

**Background and Aims:**

The long-term outcomes of percutaneous coronary intervention (PCI) vs. coronary artery bypass grafting (CABG) in patients with non-ST-segment elevation myocardial infarction (NSTEMI) and multivessel disease remain debated.

**Methods:**

The Swedish Web-system for Enhancement and Development of Evidence-based care in Heart disease Evaluated According to Recommended Therapies registry was used to analyse 57 097 revascularized patients with NSTEMI with multivessel disease in Sweden from January 2005 to June 2022. The primary endpoint was all-cause mortality, encompassing both in-hospital and long-term mortality; the secondary endpoints included myocardial infarction (MI), stroke, new revascularization, and heart failure. Multilevel logistic regression with follow-up time as a log-transformed offset variable and double-robust adjustment with the instrumental variable method were applied to control for known and unknown confounders.

**Results:**

Percutaneous coronary intervention was the primary therapy in 42 190 (73.9%) patients, while 14 907 (26.1%) received CABG. Percutaneous coronary intervention patients were generally older with more prior cardiovascular events, whereas CABG patients had higher incidences of diabetes, hypertension, left main and three-vessel disease, and reduced ejection fraction. Over a median follow-up of 7.1 years, PCI was associated with higher risks of death [adjusted odds ratio (aOR) 1.67; 95% confidence interval (CI) 1.54–1.81] and MI (aOR 1.51; 95% CI 1.41–1.62) but there was no significant difference in stroke. Repeat revascularization was three times more likely to PCI (aOR 3.01; 95% CI 2.57–3.51), while heart failure risk was 15% higher (aOR 1.15; 95% CI 1.07–1.25). Coronary artery bypass grafting provided longer survival within 15 years, especially in patients under 70 years of age, with left main disease or left ventricular dysfunction, though this benefit diminished over shorter time horizons.

**Conclusions:**

Coronary artery bypass grafting is associated with lower risks of mortality, MI, repeat revascularization, and heart failure in patients with NSTEMI, particularly in high-risk subgroups. However, its survival benefit lessens with shorter life expectancy.

## Structured Graphical Abstract

**Graphical Abstract:**
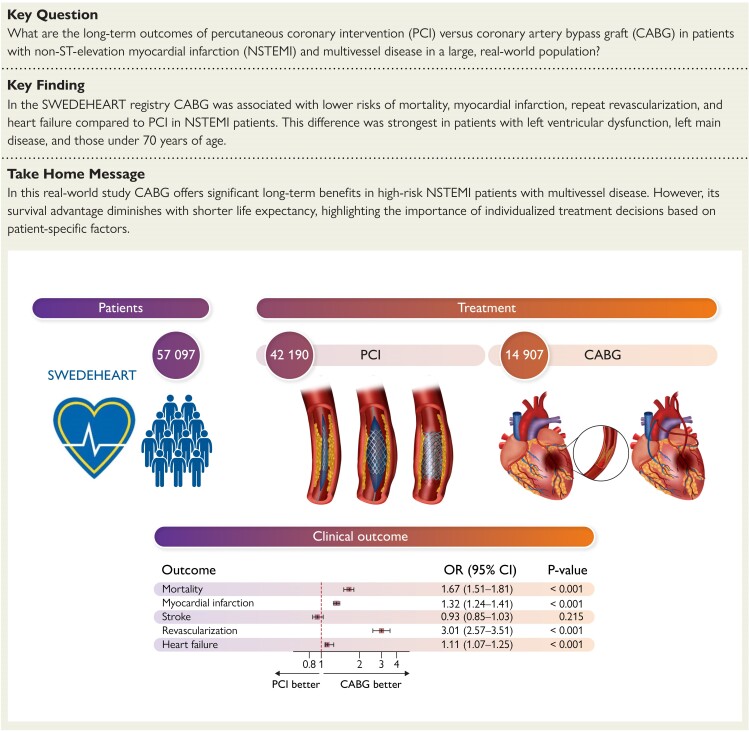
This study, using data from the Swedish Web-system for Enhancement and Development of Evidence-based care in Heart disease Evaluated According to Recommended Therapies registry, compared the long-term outcomes of coronary artery bypass grafting (CABG) and percutaneous coronary intervention in patients with non-ST-elevation myocardial infarction (NSTEMI) and multivessel disease. Based on the causal inference methods with target trial emulation and instrumental variable analysis, CABG was found to be associated with lower risks of mortality, myocardial infarction, revascularization, and heart failure, particularly in high-risk patients, including those with left ventricular dysfunction, left main disease, and those under 70 years of age. These findings underscore the importance of CABG in managing high-risk patients with NSTEMI with multivessel disease and highlight the need for individualized treatment decisions based on patient-specific characteristics and expected outcomes. CABG, coronary artery bypass grafting; CI, confidence interval; OR, odds ratio; PCI, percutaneous coronary intervention.

**See the editorial comment for this article ‘Non-STelevation acute coronary syndrome with multivessel disease: need for randomized trials', by P.C. Revaiah *et al*., https://doi.org/10.1093/eurheartj/ehae853.**

## Introduction

Observational studies are an integral part of evidence-based medicine.^1^ They offer useful information for making informed decisions in clinical practice, especially when data from randomized controlled trials (RCTs) are lacking.^2–4^

Patients with non-ST-segment elevation myocardial infarction (NSTEMI) should undergo early invasive revascularization according to the current European^5^ and American^6^ recommendations. Over the years, the adoption of percutaneous coronary intervention (PCI) has contributed to a substantial decrease in the risk of adverse cardiovascular events in NSTEMI^7^; nevertheless, coronary artery bypass grafting (CABG) remains an important treatment method for patients with NSTEMI, particularly in those with left main or complex multivessel coronary artery disease or anatomy unsuitable for PCI.

Despite the established benefits of revascularization in NSTEMI, data comparing the long-term effectiveness of PCI vs. CABG remain limited. Most large trials, such as EXCEL^8^ (Everolimus-Eluting Stents or Bypass Surgery for Left Main Coronary Artery Disease), NOBLE^9^ (Nordic-Baltic-British Left Main Revascularization), FREEDOM^10^ (Future Revascularization Evaluation in Patients with Diabetes Mellitus: Optimal Management of Multivessel Disease), and BEST^11^ (Trial of Everolimus-Eluting Stents or Bypass Surgery for Coronary Disease), either underrepresent this population or exclude patients with NSTEMI altogether, as seen in the SYNTAX^12^ (Synergy between PCI with TAXUS and Cardiac Surgery) trial. No dedicated randomized controlled trial (RCT) has compared percutaneous vs. surgical revascularization in patients with NSTEMI. Each approach has advantages and potential drawbacks, leading to ongoing discussions about their roles. Previous studies, not limited to patients with NSTEMI, suggest that CABG offers superior long-term survival and reduced incidence of major adverse cardiac events, particularly in patients with more complex coronary artery disease.^9–13^ However, PCI is less invasive and associated with shorter hospital stays with quicker recovery times, making it an attractive option for many patients and healthcare providers.

The Swedish Web-system for Enhancement and Development of Evidence-based care in Heart disease Evaluated According to Recommended Therapies (SWEDEHEART) registry offers a robust database for examining real-world outcomes of different revascularization strategies.^14^ This national registry includes detailed clinical information on patients undergoing various heart disease treatments across Sweden, enabling comprehensive analysis of treatment outcomes over extended follow-up periods.

In this study, we compared the long-term outcomes of PCI and CABG in patients with NSTEMI and multivessel disease using data from the SWEDEHEART registry. The primary endpoint was all-cause mortality, while the secondary endpoints included myocardial infarction (MI), stroke, and new revascularization. By applying statistical methods for causal inference^15,16^ and target trial emulation,^17,18^ we sought to provide insights into these two revascularization strategies’ relative benefits and risks.

## Methods

### Study design and data source

**Figure 1:**
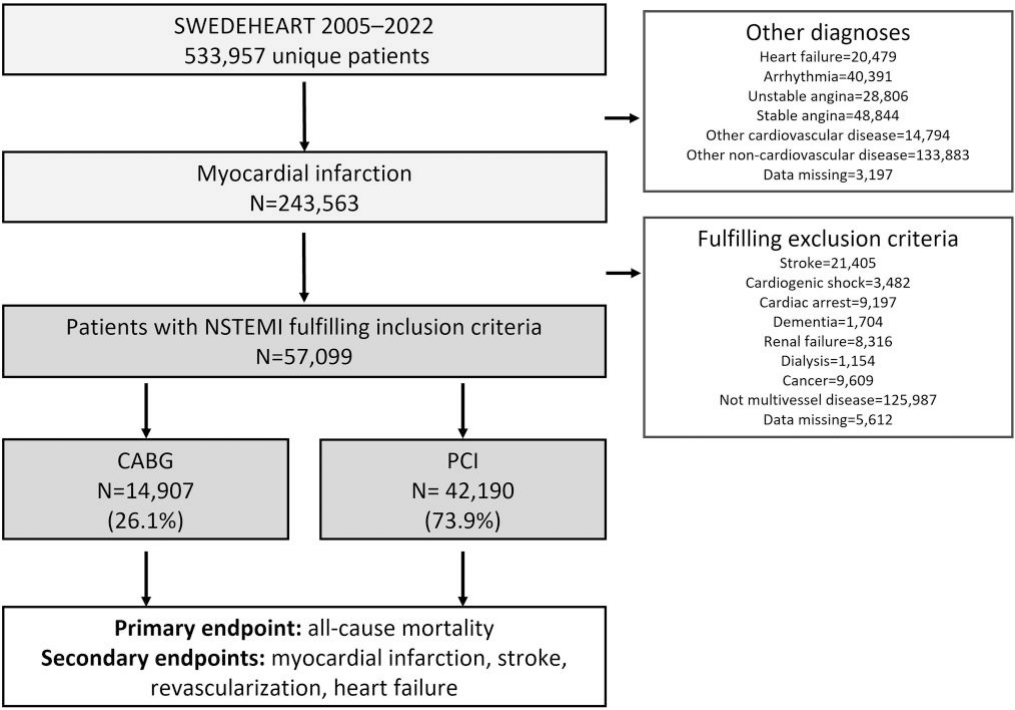
Between 2005 and 2022, 533,957 unique patients were hospitalized and reported to the registry; 243 563 (45.6%) were diagnosed with myocardial infarction, and 290 394 (54.4%) had other diagnoses. Among the patients with non-ST-elevation myocardial infarction who fulfilled the inclusion criteria, 57 097 were identified. These were further categorized into 14 907 (26.1%) who underwent coronary artery bypass grafting and 42 190 (73.9%) who underwent percutaneous coronary intervention

We conducted an observational study using data from the SWEDEHEART registry, including all revascularized patients diagnosed with NSTEMI and multivessel disease who fulfilled the inclusion criteria between January 2005 and June 2022 (*Figure 1*). The study included university hospitals with on-site cardiac surgery facilities (including CABG) and non-university hospitals that offer PCI but may refer surgical cases to nearby centres. This classification allowed for the analysis of outcomes based on hospital type.

### Study population and variables

Non-ST-elevation myocardial infarction was defined by a rise and/or fall in cardiac troponin (cTn) levels with at least one value above the 99th percentile upper reference limit and the presence of symptoms of acute myocardial ischaemia, new ischaemic electrocardiogram (ECG) changes, pathological Q waves, imaging evidence of new myocardial damage, or identification of a coronary thrombus by angiography or autopsy. The SWEDEHEART registry has adhered to the evolving Universal Definition of MI, incorporating updates from 2007, 2012, and 2018.^19^ The primary exposure of interest was the revascularization method—CABG or PCI—at the index hospitalization. Patients with unstable angina and patients who underwent both CABG and PCI were excluded. The primary outcome was all-cause mortality. All-cause mortality in this study is defined as death from any cause, encompassing both in-hospital mortality (death occurring during the initial hospitalization following the NSTEMI event) and long-term mortality (death occurring after discharge during the follow-up period). The secondary outcomes were MI, stroke, new revascularization, and heart failure. The registry collected data on demographic characteristics, comorbidities, clinical presentation, hospital characteristics, and treatment modalities. The study was approved by the Ethics Committee at the University of Gothenburg (Dnr. 759-13, date of approval 6 May 2014).

### Statistical analysis

To mitigate errors in causal inference in this observational study, we defined the analysis population using the target trial emulation method outlined by Hernán *et al*.^4,17^ and instrumental variable (IV) analysis^15^ was used to mitigate the influence of unmeasured confounders on the estimated treatment effects.

The trial emulation method involves designing our observational study protocol to mimic a hypothetical RCT by explicitly pre-specifying eligibility criteria, treatment strategies, and follow-up periods.^18^ This method helps mitigate common biases in observational studies, such as immortal time bias, selection bias, and confounding.^17^ The eligibility criteria for inclusion were NSTEMI with multivessel disease, no cardiogenic shock or cardiac arrest before admission, and no history of stroke, renal failure, cancer, or dementia, as patients with these comorbidities and single-vessel disease would be excluded from randomization to CABG in a tentative RCT.

As the primary analysis, we performed IV analysis based on the two-stage residual inclusion estimate logistic regression to account for measured and unmeasured confounders.^15^ To address confounding and ensure robust estimation of treatment effects, we employed a double-robust adjustment^20^ methodology. This approach integrates inverse probability score weighting^21^ with outcome regression modelling. Furthermore, we applied IV analysis to account for potential unmeasured confounding, which may not be fully addressed by measured covariates alone. Instrumental variable analysis is a statistical method for the causal inference of an exposure or treatment on an outcome when potentially confounding factors or unmeasured variables might bias the outcome. It involves IV, a variable that influences the treatment/exposure but is independent of the outcome. Typically, a two-stage regression model is utilized for IV analysis implementation. Initially, a regression model is fitted with the IV as the predictor variable and the treatment/exposure variable as the outcome variable. In the second stage, the predicted values of the treatment/exposure are used as the independent variable in another regression model. The outcome variable is regressed on the predicted values of the treatment/exposure while adjusting for potential confounding variables. In our investigation, the IV utilized was the quintile of preference for revascularization by PCI vs. CABG at the treating hospitals. We performed subgroup analyses to evaluate whether age, gender, diabetes, left main disease, and left ventricular (LV) ejection fraction (LVEF) modified the observed effects. We present the results of the primary IV analysis as the odds ratio for PCI vs. CABG across all available follow-ups, as well as the odds ratio for PCI vs. CABG at specific follow-up times.

Event frequencies were calculated using Kaplan–Meier estimations to analyse the time to the first event. The assumption of proportional hazards in the Cox model was violated (interaction between treatment and time, *P* < .001). Thus, logistic regression with follow-up time as a log-transformed offset variable was the primary way to compare treatments. The net treatment effect for survival was also examined using the restricted mean survival time method.^22^ Restricted mean survival time represents the average time until an outcome event occurs, calculated over a specific time horizon. It reflects the area under the survival curve up to a pre-specified time point, providing a summary measure of survival over that period. We treated hospitals as a random effect to adjust for patient-clustering within the hospitals.

Every analysis adhered to the statistical significance definition, a two-tailed *α* = .05. All statistical calculations and data visualization were performed with R (v. 4.3.1, R Foundation for Statistical Computing) and Stata (v. 18.5, StataCorp LLC). The lmtest and ivtools packages were used for two-stage residual inclusion analysis and IV validity testing. The survival and survminer packages were used for survival analyses, the gtsummary package for creating tables, and the forestploter package for creating forest plots.

Missing values in the dataset were imputed using the missRanger package in R, which utilizes random forest imputation to fill in missing values. The random forest imputation method has been shown to produce accurate imputations while preserving the data distribution, making it a reliable approach to missing data imputation.^23^ We performed sensitivity analyses to assess our findings’ robustness to the impact of residual confounding.^24,25^ For this purpose, we utilized two R packages, namely tipr and episensr. To evaluate whether treatment-induced selection bias is affecting the IV risk estimates, we applied inverse probability of selection weight.^26,27^ A placebo outcome sensitivity analysis was conducted to validate the instrument using chronic obstructive pulmonary disease as an unrelated outcome.

## Results

### Study population

The study included 57 097 patients with NSTEMI who underwent revascularization between January 2005 and June 2022. Of these patients, 42 190 (73.9%) received PCI and 14 907 (26.1%) underwent CABG. The overall cohort consisted of 75.8% men and 24.2% women, with a mean age of 68.7 ± 10.5 years. Half of the patients (50.1%) were older than 70 years.

Of the 14 907 patients who underwent CABG, 10 951 (73.5%) were initially treated at non-university hospitals and subsequently referred to university hospitals for surgery. Regarding PCI, 28.8% of the procedures were performed at university hospitals, with 12 139 out of 42 190 PCI patients treated at these centres. Overall, 28.2% of all revascularization procedures (CABG and PCI) were conducted at university hospitals, with most CABG patients being referred from non-university hospitals.

*Table 1* presents the patient characteristics. Notably, the two groups were generally balanced regarding many known risk factors, with standardized differences of ≤ .1, indicating that the groups can be considered balanced.^28^ Patients in the PCI group were, on average, 1 year older than those in the CABG group. Additionally, the PCI group had a higher prevalence of previous PCI, CABG, MI, and hypertension. Conversely, the CABG group had a higher incidence of diabetes, left main disease, three-vessel disease, Killip class > 2, and pathologic ST-T segment on ECG.

**Table 1:** Patient's characteristics at admission

Variable	*N*	CABG, *N* = 14,907^a^	PCI, *N* = 42,190^a^	Difference^b^	95% CI^b,c^
**Age**	57 097	69 (61, 75)	70 (61, 77)	−.14	−.16, −.12
**Age ≥ 70 years**	57 097			.08	.07, .10
< 70		7948 (53%)	20 730 (49%)		
≥ 70		6959 (47%)	21 460 (51%)		
**Sex**	57 097			.12	.10, .14
Male		11 844 (79%)	31 443 (75%)		
Female		3063 (21%)	10 747 (25%)		
**BMI**	50 566	27.0 (24.6, 29.8)	26.9 (24.5, 29.9)	−.01	−.03, .01
**Smoking**	56 974			.03	.01, .05
Never		5724 (39%)	16 466 (39%)		
Previous		5714 (38%)	15 894 (38%)		
Active		2930 (20%)	8142 (19%)		
**COPD**	57 097	779 (5.2%)	2202 (5.2%)	.00	−.02, .02
**Myocardial infarction**	57 097	1327 (8.9%)	5600 (13%)	−.14	−.16, −.12
**Angina pectoris**	57 097	4161 (28%)	7252 (17%)	.26	.24, .28
**Diabetes**	57 097	4194 (28%)	9647 (23%)	.12	.10, .14
**Hyperlipidaemia**	57 046	4914 (33%)	13 484 (32%)	.03	.01, .04
**PAD**	57 097	567 (3.8%)	1641 (3.9%)	.00	−.02, .01
**Hypertension**	57 097	3184 (21%)	10 930 (26%)	−.11	−.13, −.09
**Prior CABG**	57 097	95 (.6%)	4111 (9.7%)	−.42	−.44, −.40
**Prior PCI**	57 097	747 (5.0%)	4033 (9.6%)	−.18	−.19, −.16
**Prior bleeding**	57 097	375 (2.5%)	1262 (3.0%)	−.03	−.05, −.01
**Heart failure**	57 097	738 (5.0%)	2191 (5.2%)	.01	−.01, .03
**Ejection fraction**	47 571			.06	.04, .08
≥ 50%		9042 (68%)	23 552 (69%)		
40%–49%		2373 (18%)	6085 (18%)		
30%–39%		1339 (10%)	2924 (8.5%)		
< 30%		519 (3.9%)	1281 (3.7%)		
**ECG rhythm**	56 857			.06	.04, .08
Sinus		13 761 (93%)	38 441 (92%)		
AFib/Aflutter		874 (5.9%)	2675 (6.4%)		
Other		164 (1.1%)	761 (1.8%)		
**ECG QRS-complex annotation**	56 672			.09	.07, .11
Normal		10 445 (71%)	29 604 (71%)		
Pacemaker		80 (.5%)	420 (1.0%)		
LBBB		586 (4.0%)	1993 (4.8%)		
Q-wave		1396 (9.5%)	3407 (8.1%)		
RBBB		620 (4.2%)	2014 (4.8%)		
Other		1475 (10.0%)	3916 (9.3%)		
**ECG ST-T changes**	56 737			.16	.14, .17
Normal		4522 (31%)	14 742 (35%)		
ST-depression		5550 (38%)	13 140 (31%)		
Pathologic T-wave		2351 (16%)	6203 (15%)		
Other		1723 (12%)	5399 (13%)		
**Heart rate**	55 278	77 (66, 90)	75 (65, 88)	.10	.08, .12
**Systolic blood pressure**	54 978	153 (137, 170)	153 (137, 170)	.00	−.02, .02
**Diastolic blood pressure**	53 872	85 (76, 96)	86 (76, 97)	−.01	−.03, .01
**Pulmonary rales**	55 744			.06	.04, .08
No		13 147 (91%)	38 041 (92%)		
Basal rales		831 (5.8%)	2227 (5.4%)		
Rales > ½ lungs		110 (.8%)	200 (.5%)		
Lung oedema		184 (1.3%)	349 (.8%)		
**Killip ≥ 2**	55 165	2428 (17%)	6388 (16%)	.04	.02, .06
**Revascularization timing**	52 289			−.10	−.07, −.13
Elective		2544 (19%)	5562 (14%)		
Subacute		8085 (62%)	24 466 (62%)		
Acute		2505 (19%)	9127 (23%)		
**Severity of CAD**	56 178			.71	.69, .73
2-VD		1642 (11.3%)	25 237(59.9)		
3-VD		7459 (51.5%)	13 000 (30.9%)		
LM		5388 (37.2%)	3874 (9.2%)		
**Glucose (mmol/L)**	48 840	6.6 (5.7, 8.6)	6.6 (5.7, 8.4)	.04	.02, .06
**Total cholesterol (mmol/L)**	45 611	5.00 (4.20, 5.90)	5.00 (4.10, 5.90)	.03	.01, .05
**Triglycerides (mmol/L)**	42 543	1.43 (1.10, 2.00)	1.40 (1.10, 2.00)	.01	−.01, .03
**HDL cholesterol (mmol/L)**	44 472	1.10 (.92, 1.36)	1.10 (.94, 1.40)	−.05	−.08, −.03
**LDL cholesterol**	43 588	3.11 (2.32, 3.91)	3.06 (2.30, 3.86)	.05	.03, .07
**Creatinine (mmol/L)**	55 430	82 (70, 95)	81 (70, 95)	−.02	−.04, .00
**CRP (g/L)**	50 517	5 (2, 9)	5 (2, 9)	.01	−.01, .03
**Haemoglobin (mg/L)**	49 782	143 (133, 152)	143 (132, 152)	.05	.03, .07
**Beta-blocker**	57 069	5085 (34%)	15 165 (36%)	.04	.02, .06
**ACEi/ARBi**	56 377			.05	.03, .07
No		9368 (64%)	26 168 (63%)		
ARBi		2279 (16%)	7158 (17%)		
ACEi		2945 (20%)	8068 (19%)		
ARBi + ACEi		109 (.7%)	282 (.7%)		
**Calcium antagonist**	57 069	3098 (21%)	8742 (21%)	.01	−.01, .03
**Digitalis**	57 069	139 (.9%)	498 (1.2%)	.03	.01, .05
**Diuretics**	57 069	2590 (17%)	7726 (18%)	.03	.01, .05
**Statins**	57 069	4783 (32%)	13 042 (31%)	.03	.01, .05
**Other lipid-lowering**	41 749			.04	.02, .06
No		11 148 (97%)	29 525 (97%)		
Ezetrol		110 (1.0%)	361 (1.2%)		
Fibrates		35 (.3%)	125 (.4%)		
Other		41 (.4%)	98 (.3%)		
**Nitrates**	57 069	1504 (10%)	4283 (10%)	.01	−.01, .03
**Diabetes medications**	56 489			.09	.07, .11
No		11 635 (79%)	34 419 (82%)		
Tablets		1584 (11%)	3575 (8.6%)		
Insulin		827 (5.6%)	1972 (4.7%)		
Insulin + tablets		694 (4.7%)	1783 (4.3%)		
**Oral anticoagulants**	57 307			.08	.06, .09
No		14 145 (95%)	39 684 (94%)		
Warfarin		414 (2.8%)	1476 (3.5%)		
Dabigatran		23 (.2%)	84 (.2%)		
Rivaroxaban		55 (.4%)	160 (.4%)		
Apixaban		96 (.6%)	503 (1.2%)		
Edoxaban		0 (0%)	8 (<.1%)		
Other		0 (0%)	2 (<.1%)		
**ASA**	57 307	5286 (35%)	14 885 (35%)	.01	.00, .03
**Other antiplatelets**	57 307			.05	.03, .07
No		14 139 (95%)	39 853 (94%)		
Clopidogrel		508 (3.4%)	1765 (4.2%)		
Ticlopidine		1 (<.1%)	4 (<.1%)		
Prasugrel		1 (<.1%)	7 (<.1%)		
Ticagrelor		43 (.3%)	184 (.4%)		
Other		42 (.3%)	102 (.2%)		

2-VD, two-vessel disease; 3-VD, three-vessel disease; ACEi, angiotensin-converting enzyme inhibitor; AFib, atrial fibrillation; ARBi, angiotensin II receptor blocker; ASA, acetylsalicylic acid; BMI, body mass index; CABG, coronary artery bypass grafting; CAD, coronary artery disease; COPD, chronic obstructive pulmonary disease; CRP, C-reactive protein; ECG, electrocardiogram; HDL, high-density lipoprotein; LBBB, left bundle branch block; LM, left main; PAD, peripheral artery disease; PCI, percutaneous coronary intervention; RBBB, right bundle branch block.

^a^Median [interquartile range (IQR)]; *n* (%).

^b^Standardized mean difference.

^c^CI, confidence interval.

### Treatment trends

**Figure 2:**
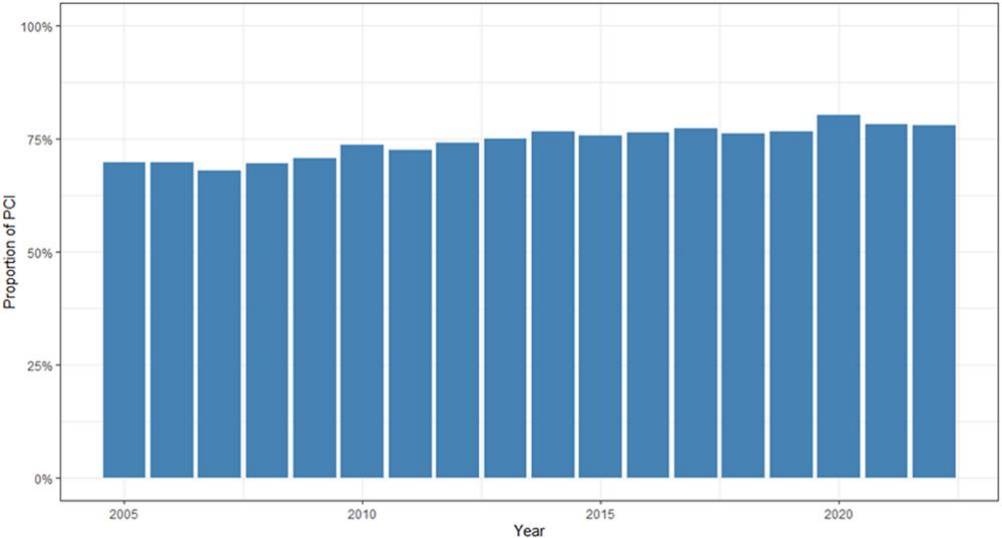
Trends in the proportion of percutaneous coronary intervention as the method of choice for revascularization of patients with non-ST-elevation myocardial infarction in Sweden from 2005 to 22. The bar chart depicts the yearly percentage of patients undergoing percutaneous coronary intervention, illustrating a steady increase in its utilization since 2005

**Figure 3:**
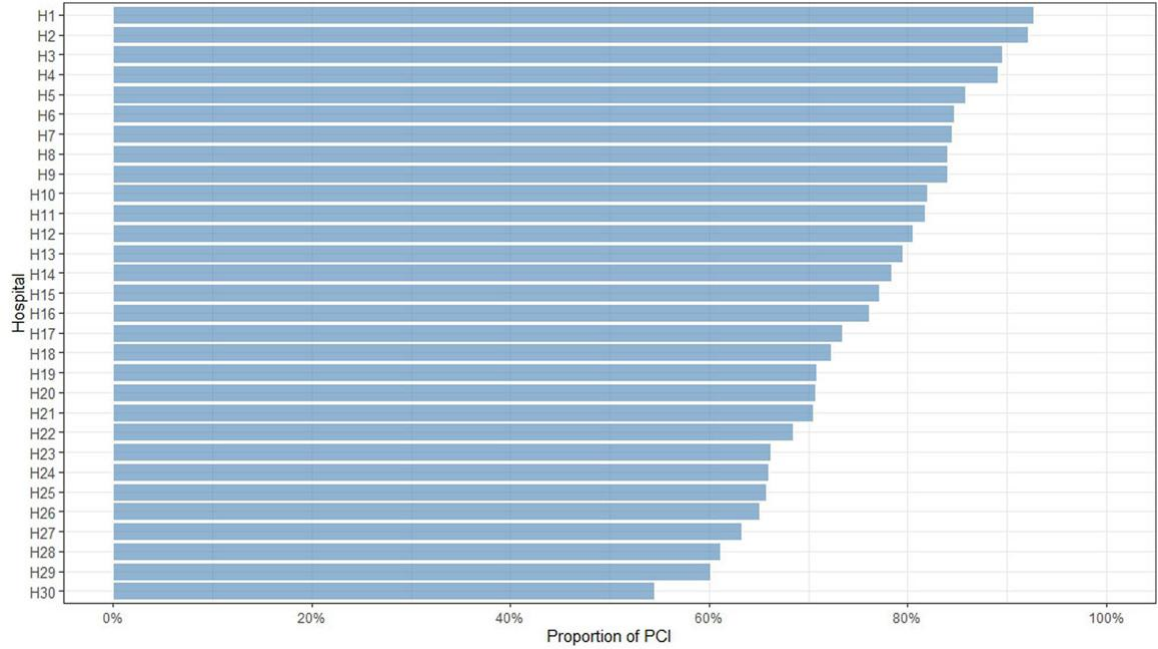
The considerable variation in the proportion of percutaneous coronary intervention utilization across different hospitals in Sweden. The use of percutaneous coronary intervention varied significantly, ranging from 55% in some hospitals to nearly 92% in others. This considerable variation indicates differences in the preference and possibly the availability of percutaneous coronary intervention to revascularize patients with non-ST-elevation myocardial infarction among hospitals. This substantial variation indicates a lack of standardization in using coronary artery bypass grafting and percutaneous coronary intervention for managing patients with non-ST-elevation myocardial infarction in Swedish hospitals

Over the study period, the use of PCI for revascularization increased annually by 3.4% [(*P*_trend_ < .001), *Figure 2*]. The preference for PCI varied significantly between hospitals, with rates ranging from 55% to 92% [(*P* < .001), *Figure 3*].

#### Instrumental variable analysis

Our IV analysis demonstrated that the instrument was valid and relevant, passing the under-identification test (*P* < .001), the overidentification test (*P* = .358), and the weak identification test (Cragg–Donald Wald F statistic = 669, *P* < .001). In the first stage of our IV analysis, we identified a significant association between the treatment-preference instrument and the choice of revascularization method (*P* < .001). In the second stage of the analysis, we observed a 67% increase in mortality linked to PCI use (*Table 2*, *P* < .001).

**Table 2:** Risk estimates in different statistical models

Model	OR	95% CI	*P*-value
**Mortality**			
Unadjusted logistic regression	1.18	1.13–1.23	<.001
Multivariable logistic regression^a^	1.59	1.49–1.71	<.001
*IV logistic regression^b^	1.67	1.54–1.81	<.001
**Myocardial infarction**			
Unadjusted logistic regression	1.45	1.37–1.54	<.001
Multivariable logistic regression^a^	1.38	1.29–1.48	<.001
IV logistic regression^b^	1.51	1.41–1.62	<.001
**Stroke**			
Unadjusted logistic regression	.94	.87–1.12	.140
Multivariable logistic regression^a^	.96	.87–1.06	.401
IV logistic regression^b^	.93	.85–1.03	.215
**Revascularization**			
Unadjusted logistic regression	3.23	3.04–3.44	<.001
Multivariable logistic regression^a^	3.35	3.08–3.65	<.001
IV logistic regression^b^	3.01	2.57–3.51	<.001
**Heart failure**			
Unadjusted logistic regression	1.10	1.05–1.16	<.001
Multivariable logistic regression^a^	1.19	1.12–1.28	<.001
IV logistic regression^b^	1.15	1.07–1.25	<.001

The reference group in all models is PCI.

ECG, electrocardiogram, IV, instrumental variable.

^a^Multivariable logistic regression with follow-up time as a log-transformed offset variable with the following covariates: age at admission, sex, smoking status, history of chronic obstructive pulmonary disease, heart failure, history of myocardial infarction, diabetes mellitus, peripheral arterial disease, hypertension, coronary artery bypass grafting, percutaneous coronary intervention, body mass index, Killip ≥ 2, ST-T changes, ECG rhythm.

^b^Instrumental variable analysis with treatment-preference instrument based on the quintiles of the hospital preference for using percutaneous coronary intervention. The IV model also used all the covariates from the multivariable logistic regression. *Primary analysis.

### Outcomes

The median follow-up time was 7.1 years, ranging from 1 day to 17.1 years. During this period, there were 17 731 deaths (31.1%), 12 765 myocardial infarctions (22.4%), and 4833 stroke events (8.5%). The outcome data from the different statistical models are presented in *Table 2*.

#### All-cause mortality

**Figure 4:**
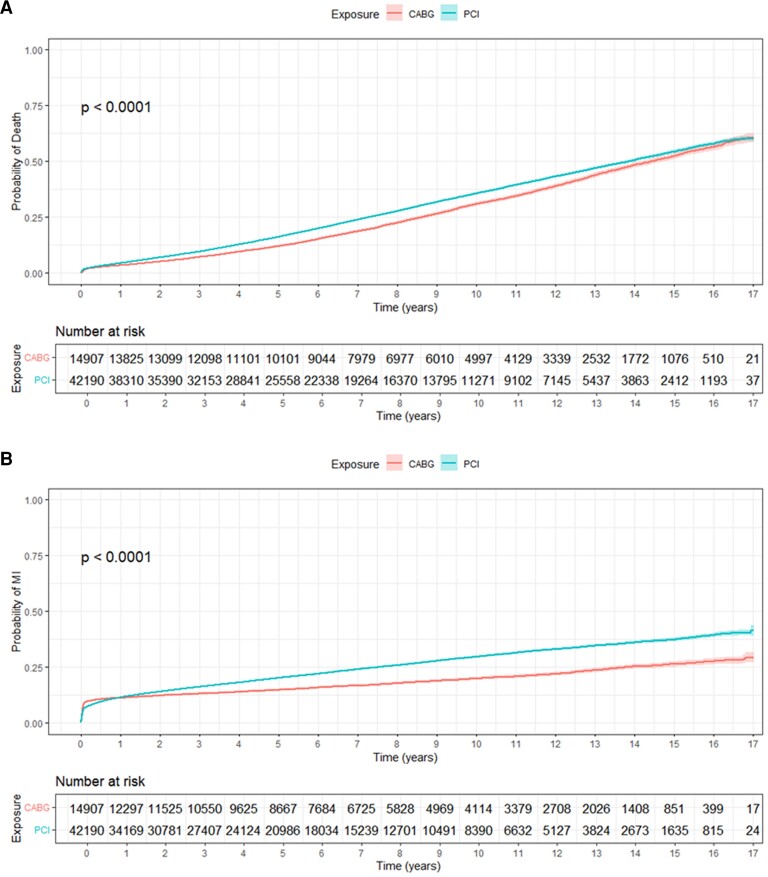

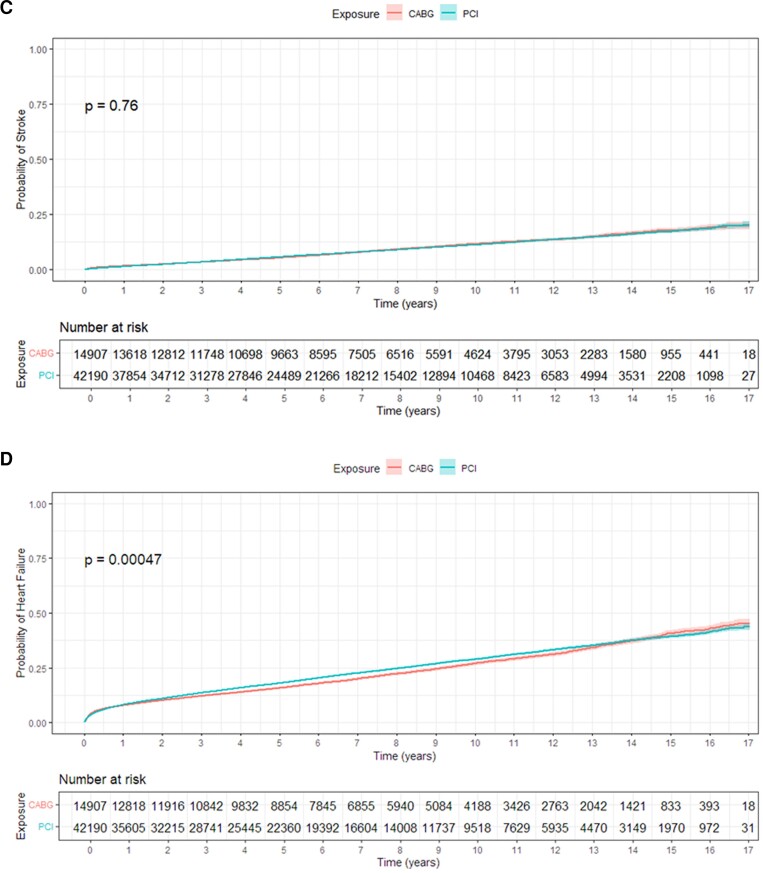
Kaplan–Meier curves for all-cause mortality in patients with CS. (*A*) All-cause mortality in patients revascularized with coronary artery bypass grafting and percutaneous coronary intervention. (*B*) Risk of myocardial infarction. (*C*) Risk of stroke. (*D*) Risk of heart failure

Patients who underwent PCI had a 67% higher adjusted odds ratio (aOR) for all-cause mortality compared with those who underwent CABG [aOR 1.67; 95% confidence interval (CI) 1.54–1.81; *P* < .001, *Figure 4A*]. Analyses of the restricted mean survival time showed that the benefit of CABG increased gradually, starting 5 years after revascularization (see Supplementary data online, *Figure S1*, left panel). For patients who lived at least 15 years after revascularization, the average lifetime was 6.7 months (95% CI 4.9–8.4) longer after CABG (*P* < .001). However, for patients who lived ≤ 5 years, the average lifetime was 25 days (95% CI 18.0–32.4, *P* < .001) longer after CABG (see Supplementary data online, *Figure S1*, right panel). Of the patient cohort, 3488 (6.1%) individuals had a survival time of at least 15 years, while 35 659 (84.9%) had a survival time of at least 5 years during the study period. The mortality benefit of CABG over PCI was evident at each yearly follow-up interval. Coronary artery bypass grafting consistently showed a lower risk of all-cause mortality than PCI across the entire 10-year period (*Table 3*).

**Table 3:** Odds ratio for mortality after percutaneous coronary intervention vs. coronary artery bypass grafting at different lengths of follow-up

Mortality	OR^a^	95% CI	*P*-value
1 year	1.75	1.53–2.01	<.001
2 years	1.59	1.49–1.71	<.001
3 years	1.67	1.54–1.81	<.001
4 years	1.62	1.47–1.79	<.001
5 years	1.64	1.50–1.81	<.001
6 years	1.59	1.45–1.74	<.001
7 years	1.54	1.40–1.68	<.001
8 years	1.50	1.38–1.65	<.001
9 years	1.47	1.34–1.62	<.001
10 years	1.40	1.27–1.55	<.001

CI, confidence interval; OR, odds ratio.

^a^Instrumental variable analysis with treatment-preference instrument based on the quintiles of the hospital preference for using percutaneous coronary intervention. The IV model also used all the covariates from the multivariable logistic regression.

#### Myocardial infarction

The risk of MI for the whole follow-up period was higher in the PCI group, with 51% increased risk compared with the CABG group [(aOR 1.51; 95% CI 1.41–1.62; *P* < .001), *Figure 4B*]. However, the risk of MI was higher in the CABG group up to 1 year after revascularization (aOR 1.31; 95% CI 1.21–1.41; *P* < .001).

##### Stroke

There was no significant difference in the risk of stroke between the two groups [(aOR .93; 95% CI .85–1.03; *P* = .215), *Figure 4C*].

#### Revascularization

The likelihood of repeated revascularization was three times higher in the PCI group (aOR 3.01; 95% CI 2.57–3.51; *P* < .001).

#### Heart failure

The risk of heart failure for the whole follow-up period was higher in the PCI group, with a 15% increased risk compared with the CABG group [(aOR 1.15; 95% CI 1.07–1.25; *P* < .001), *Figure 4D*].

### Subgroup analysis

**Figure 5:**
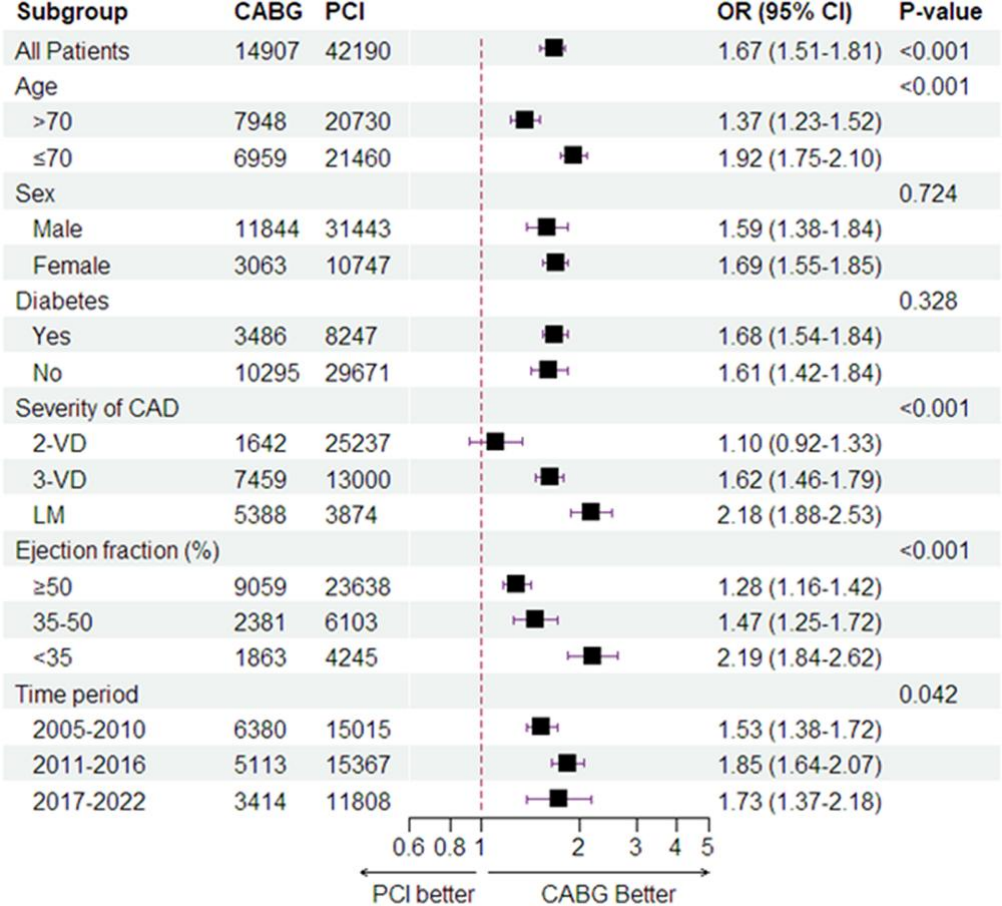
A subgroup analysis for age, sex, diabetes, presence of left main disease, and ejection fraction. The data are presented as a forest plot, which summarizes the odds ratios and 95% confidence intervals of the different subgroups on mortality. The results of the subgroup analysis indicated that the effect of revascularization with coronary artery bypass grafting varied across some subgroups. There was evidence for quantitative interaction with a higher risk with percutaneous coronary intervention adults < 70 years, patients with left main disease, and patients with lower ejection fraction. There was no interaction between the revascularization methods and sex and diabetes

Our subgroup analyses revealed significant effect modification. Coronary artery bypass grafting showed a more pronounced survival benefit in patients with LV dysfunction, severe coronary artery disease, and those younger than 70 years (all *P*_interaction_ < .001). No significant interaction was found between the revascularization method and diabetes or sex (*Figure 5*). The interaction test revealed a statistically significant variation in all-cause mortality trends over time (*P* = .042), with CABG consistently showing a survival advantage over PCI across all periods (*Figure 5*). Specifically, the survival benefit of CABG was most pronounced during 2011–22. Additionally, we observed no interaction between the types of hospital (high-volume university hospitals with on-site CABG vs. other hospitals). Specifically, the analysis of PCI outcomes across hospital types showed no significant difference (*P* = .973) for PCI performed at university hospitals compared with non-university hospitals. Furthermore, no significant interaction was found between the revascularization method and peripheral arterial disease (*P*_interaction_ = .246).

### Sensitivity analysis

The sensitivity analyses were performed to quantify the amount of residual confounding to negate the estimated adjusted risk estimates and have yielded the following results.

#### Mortality

To negate the estimated aOR of 1.67, unmeasured confounders would need to be present in 80% of the PCI group and 20% of the CABG group, with an odds ratio (OR) of 2.49 between these confounders and mortality.

#### Myocardial infarction

To negate the estimated aOR of 1.32, unmeasured confounders would need to be present in 60% of the PCI group and 30% of the CABG group, with an OR of 2.57 between these confounders and MI.

#### Revascularization

To negate the estimated aOR of 3.01, unmeasured confounders would need to be present in 80% of the PCI group and 20% of the CABG group, with an OR of 11.2 between these confounders and revascularization.

#### Heart failure

To negate the estimated aOR of 1.15, unmeasured confounders would need to be present in 80% of the PCI group and 20% of the CABG group, with an OR of 1.26 between these confounders and revascularization.

After applying inverse probability of selection weights, the estimated risk from the IV models remained substantially unchanged for mortality (OR 1.59; 95% CI 1.43–1.77, *P* < .001), for MI (OR 1.27; 95% CI 1.16–1.41, *P* < .001), and for stroke (OR .93; 95% CI .82–1.03, *P* = .188). These results support the instrument’s validity, implying that it is not associated with treatment-induced selection bias. The placebo outcome sensitivity analysis based on the IV model found no significant association between the instrument and post-discharge hospitalization for chronic obstructive pulmonary disease (OR 1.05; 95% CI .94–1.22, *P* = .271). This lack of association supports the validity of our IV, as it indicates that the instrument does not affect the outcome unrelated to the treatment.

## Discussion

We used trial emulation and IV analysis to examine the relationship between revascularization methods and long-term mortality in a cohort of 57 097 patients with NSTEMI and multivessel disease in the SWEDEHEART registry. Our findings indicate that CABG is associated with lower risks of all-cause mortality and MI compared with PCI. Specifically, the long-term risk of all-cause mortality was 41% lower in the CABG group, and the risk of MI was 34% lower. The mortality benefit of CABG over PCI was evident at each yearly follow-up interval. Conversely, the likelihood of repeated revascularization was three times higher in the PCI group. There was no significant difference in the risk of stroke between the two groups (*Structured Graphical Abstract*).

The survival benefit of CABG was more pronounced in specific subgroups, including patients younger than 70 years, those with left main disease, and individuals with LV dysfunction. This aligns with evidence suggesting that patients with more complex coronary artery disease benefit more from surgical revascularization.^29–31^ Our findings further reinforce that CABG offers significantly greater benefits in patients with reduced LVEF, particularly those with LVEF < 35%, likely due to better protection against future ischaemic events. This suggests that LVEF should play a significant role in selecting revascularization strategies, as patients with severely reduced LVEF may derive a greater survival advantage from CABG. In contrast, for patients with preserved or moderately impaired LVEF, the benefits of CABG are less pronounced relative to the associated risks. In these cases, the decision should be guided by other factors such as anatomical complexity, comorbidities, and patient preferences. This highlights the importance of a personalized approach to treatment, considering LVEF and other risk factors to optimize the balance between risks and benefits.

The optimal management approach for patients with NSTEMI and multivessel disease concerning long-term clinical outcomes remains uncertain, as no dedicated RCTs have addressed this issue. Our findings are consistent with several previous studies and meta-analyses that have demonstrated the superiority of CABG over PCI in terms of long-term survival and reduced incidence of major adverse cardiac events in patients with complex coronary artery disease.^9,12,29,30^

Myocardial infarction is the primary cause of death in patients with coronary artery disease, and the significant reduction in MI risk with CABG fits well with the decreased risk of death. Coronary artery bypass grafting resolves ischaemia by bypassing the flow-limiting lesions in coronary arteries but also offers protection against the future consequences of plaque rupture proximal to the anastomosed vessels via ‘surgical collateralization.’^32^ However, the risk of MI was higher in CABG during the initial period post-revascularization. This finding is supported by the data from previous RCTs, where early complications, including procedure-related MI, were lower in the PCI arm.

The current study, encompassing 57 097 patients, is the most extensive comparative analysis of CABG and PCI in patients with NSTEMI. This comprehensive patient population significantly exceeds the 13 observational studies analysed in the recent meta-analysis, which included 48 891 patients.^33^ While the revascularization results are consistent, their analysis did not find a significant difference in death and MI between PCI and CABG. Shorter follow-up periods, fewer events, and limited statistical power could explain the discrepancy. Our study’s large sample size, with numerous clinical events over a follow-up period of up to 17 years, provides robust statistical power. This extensive data set enables more precise subgroup analyses and a thorough investigation of secondary endpoints, addressing many limitations in smaller studies. While PCI offers the advantages of being less invasive, with shorter hospital stays and quicker recovery times, CABG provides significant long-term survival benefits, particularly for patients with specific high-risk features. Coronary artery bypass grafting is associated with a lower risk of death and, on average, adds ∼6 months of survival time over 15 years. This is a substantial survival benefit compared with other well-established life-prolonging treatments in medicine. Notably, the survival benefit of CABG should not be interpreted as an increase in life span that occurs only in the future; instead, a gain in life expectancy implies a potential immediate and continuous benefit.^34,35^ However, the survival benefit of CABG diminishes significantly with shorter life expectancy. The survival benefit is reduced to an average of 25 days for patients with an anticipated life expectancy of 5 years. While 6 months represents a substantial gain, 25 days is relatively modest. Heart teams should carefully consider this information when making treatment recommendations to patients. Our study highlights the necessity of individualized treatment plans, particularly given the substantial effect modification observed in patients with LV dysfunction, left main disease, and those younger than 70 years. Involving patients in decision-making is essential to align treatment choices with their preferences and values.

This study highlights the need for further research to refine revascularization strategies in patients with NSTEMI and multivessel disease. RCTs with CABG are warranted to compare contemporary PCI techniques, including newer-generation drug-eluting stents and advanced imaging modalities. Advances in diagnostic techniques have enabled the detection of minor non-fatal cardiovascular events, often neither associated with symptoms nor affecting quality of life, highlighting the uncertainty in defining clinically relevant non-fatal events and how to account for the competing risk of death. Additionally, exploring the role of hybrid revascularization approaches and personalized medicine strategies could provide valuable insights into optimizing treatment for this complex patient population. Future trials should also focus on a broader range of outcomes, including renal, pulmonary, neuropsychological, and quality-of-life measures. They should consider recurrent events rather than just time-to-first-event analysis. This approach will allow for accurately informed treatment decisions based on clinical status and personal expectations and goals to be made by individual patients and their treating physicians.

Several RCT studies,^36^ but not all (e.g. SYNTAX,^12^ EXCEL,^8^ and NOBLE^9^), have demonstrated that patients with diabetes often derive significantly more long-term benefits from CABG than PCI. In our study, the lack of significant differences in outcomes between CABG and PCI for patients with diabetes may be attributed to several factors, including the specific characteristics and selection criteria of our real-world patient population, which may have resulted in a cohort where the advantages of CABG were less evident. Additionally, advancements in PCI techniques and improvements in the medical management of diabetes in Sweden^37^ over the study period likely contributed to narrowing the outcome differences between the two revascularization strategies.

One of the primary strengths of this study is the use of the SWEDEHEART registry, which provides comprehensive data encompassing a real-world patient population from one whole nation. The application of advanced statistical methods, including IV analysis^15^ and double-robust adjustment, following the recommendation for target trial emulation,^4,17^ enhances the validity of the findings by addressing the weaknesses of observational studies, such as measured and unmeasured confounders and immortality bias. However, several limitations should be acknowledged. First, the study’s observational nature limits the ability to establish definitive causality. Despite rigorous adjustment for confounders, residual confounding cannot be entirely ruled out. However, our formal sensitivity analyses, which quantify the amount of residual confounding needed to nullify the risk estimates, indicate the robustness of our results. The extended duration of our study, spanning over 17 years, offers valuable insights into long-term outcomes. However, it also presents certain challenges, particularly regarding changes in clinical practice, PCI technology (e.g. drug-eluting stents), CABG techniques, and post-procedural medical management. These advances may have impacted outcomes in both groups and should be considered when interpreting our results. Second, we could not adjust for the prevalence of chronic total occlusion, completeness of revascularization, and compliance with post-discharge drugs. We did not have information on Type-2 MI, peri-procedural MI, or the use of mechanical assist devices. Peri-procedural MI may affect short-term outcomes (such as length of hospital stay, use of mechanical circulatory support, and peri-operative complications) and long-term outcomes (such as survival, heart failure, and repeat revascularization). Furthermore, the lack of information on the cause or association between peri-procedural MI and adverse long-term outcomes limits our ability to explore these relationships comprehensively. We acknowledge that our study did not capture a comprehensive range of peri-procedural complications commonly associated with CABG, such as low cardiac output syndrome, atrial fibrillation, acute kidney injury, wound infections, respiratory failure, bleeding requiring re-exploration, and related fatalities. This limits our ability to provide a fuller picture of the broader peri-operative risks, which is crucial to understanding patient outcomes following CABG. Another limitation is the lack of detailed information on the types of grafts used in CABG procedures, including the distinction between arterial and vein grafts. Additionally, we did not have data on the number, type, or dimensions of stents used in PCI procedures. We did not have access to the SYNTAX score in our database, which limits our ability to assess the complexity of coronary artery disease in more detail and its potential impact on treatment outcomes. This absence may affect the granularity of our analysis, and future studies incorporating SYNTAX scores could provide more detailed insights. We could not specifically assess the impact of peripheral arterial disease on peri-procedural complication rates in patients undergoing PCI or CABG. While peripheral arterial disease is known to influence complications during these procedures, our study did not evaluate this aspect. However, we did not observe any effect modification between peripheral arterial disease and the primary or secondary outcomes. Finally, our study was performed in Swedish hospitals, and the results may not be valid for other countries with different healthcare systems, treatment, and diagnostic practices.

## Conclusions

This study provides evidence that CABG is associated with lower mortality, MI, revascularization, and heart failure risks than PCI in patients with NSTEMI and multivessel disease. The survival benefit with CABG is substantially decreased when life expectancy is < 5 years. These findings support the use of CABG in patients with NSTEMI and emphasize the importance of individualized treatment decisions based on patient-specific characteristics and expected outcomes.

## Supplementary data

Supplementary data are available at *European Heart Journal* online.

## Supplementary Material

ehae700_Supplementary_Data

## Declarations

### Data Availability

Data from this study will not be publicly available following Swedish laws protecting the privacy of research participants.
