# Supplementary material for: Percutaneous vs. surgical revascularization of non-ST-segment elevation myocardial infarction with multivessel disease: the SWEDEHEART registry
Source: Eur Heart J. 2024 Nov 27;46(6):518–31. doi: 10.1093/eurheartj/ehae700 (PMC11804248; doi:10.1093/eurheartj/ehae700)
Supplement: ehae700_Supplementary_Data [file ehae700_supplementary_data.docx]

**Supplemental Figure 1**


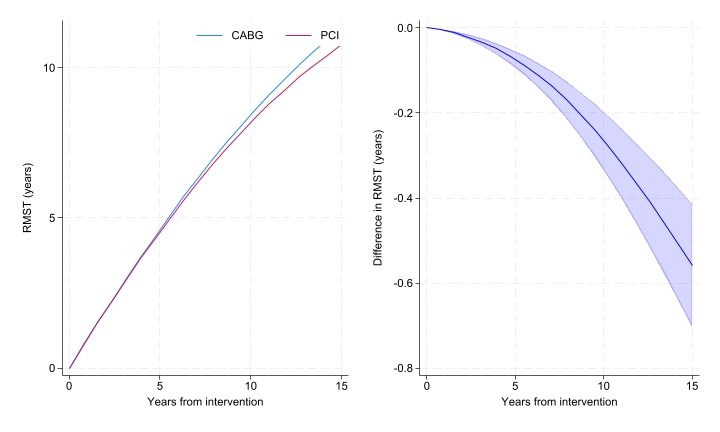


Left panel: Analyses of the restricted mean survival time (RMST) show that the survival benefit of coronary artery bypass grafting (CABG) over percutaneous coronary intervention (PCI) becomes increasingly apparent starting five years after revascularization.

Right panel: The difference in RMST between CABG and PCI over time is shown. For patients who lived five years or less, the average lifetime was 25 days longer after CABG (95% CI 18.0-32.4, P < 0.001). For patients who survived up to 15 years after revascularization, the average lifetime was 6.7 months longer after CABG (95% CI 4.9-8.4, P < 0.001). The shaded area represents the 95% confidence intervals for the difference in RMST, showing the growing survival advantage of CABG as the follow-up period extends.
